# Supplementary material for: Male and Female Mitochondria Respond Differently after Exercising in Acute Hypoxia
Source: Biomedicines. 2023 Nov 26;11(12):3149. doi: 10.3390/biomedicines11123149 (PMC10740434; doi:10.3390/biomedicines11123149)
Supplement: Supplementary file 1 [file biomedicines-11-03149-s001.zip › Legend Supplemental material.pdf]

Legends of Supplemental material

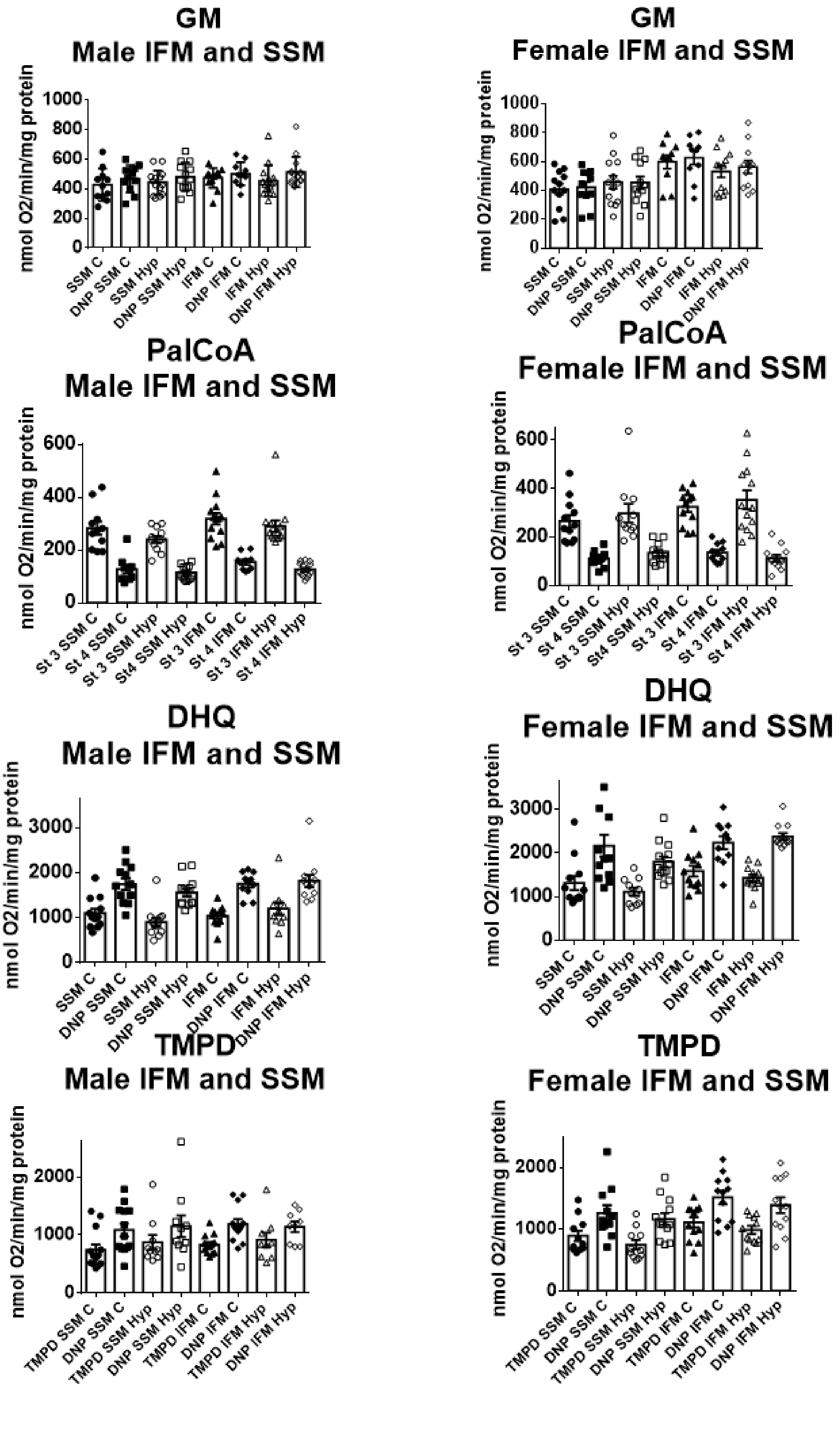

Figure S1. Graphs show the OXPHOS values of hypoxic and normoxic IFM and SSM in male and female rats. The following substrates were used: Glutamate and malate (GM, complex I), palmitoyl CoA (lipid substrate), DHQ (complex III), TMPD (complex IV). OXPHOS rates after TMPD administration have been subtracted by the oxygen consumption not of complex IV origin, evidenced by the azide inhibition of the latter. DNP (dinitrophenol) is an uncoupling agent, testing the maximum ETS efficiency. None of the data presented a statistically significant difference with control values. Data are means ± SEM, n=10-14.

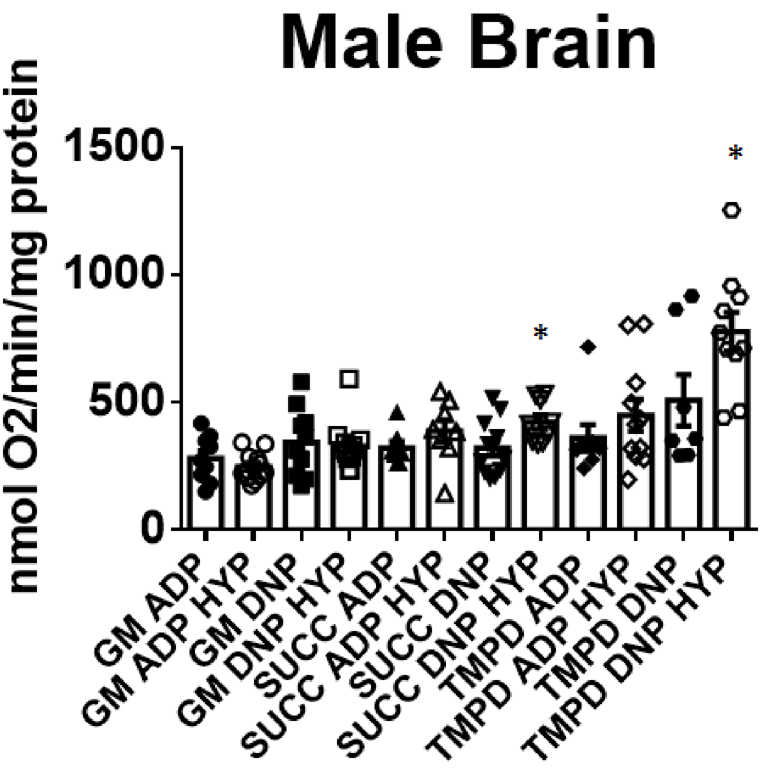

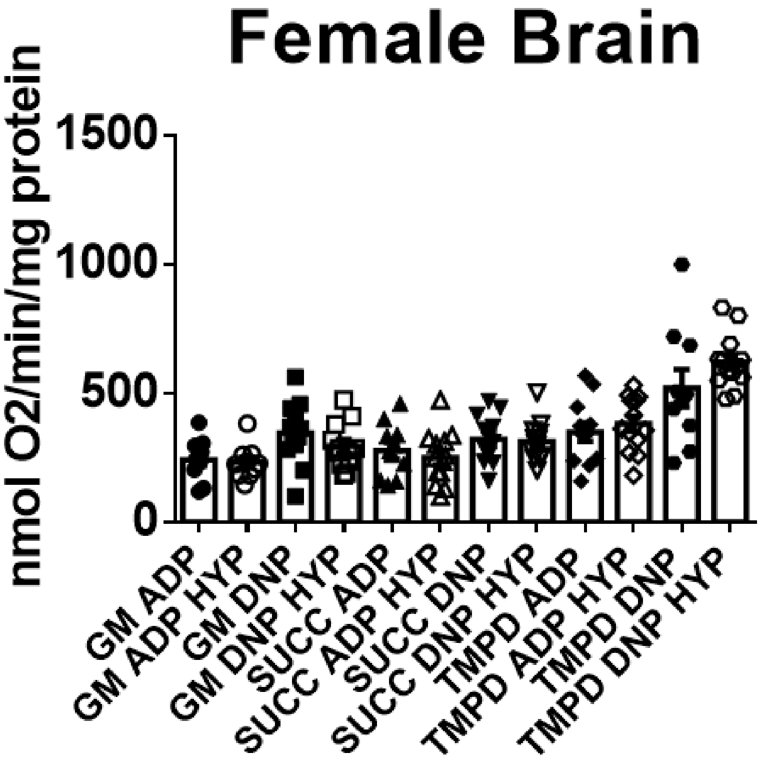

Figure S2. OXPHOS rates of hypoxic and normoxic brain mitochondra in male and female rats. The following substrates were used: Glutamate and malate (GM, complex I),Succinate and Rotenone (Succ, complex II), TMPD (complex IV). OXPHOS rates after TMPD administration have been subtracted by the oxygen consumption not of complex IV origin, evidenced by the azide inhibition of the latter. DNP (dinitrophenol) is an uncoupling agent, testing the maximum ETS efficiency. Data are means ± SEM, n=10-13.
